# Allelopathic Potential of Invasive *Plantago virginica* on Four Lawn Species

**DOI:** 10.1371/journal.pone.0125433

**Published:** 2015-04-27

**Authors:** Huatian Wang, Yumei Zhou, Yang Chen, Quanxi Wang, Lifen Jiang, Yiqi Luo

**Affiliations:** 1 Shanghai Normal University, Shanghai, China; 2 Shanghai Institute of Technology, Shanghai, China; 3 University of Oklahoma, Norman, Oklahoma, United States of America; Shandong University, CHINA

## Abstract

*Plantago virginica* L. has invaded many lawn ecosystems in the Eastern part of China. The invasion has incurred an economic cost to remove them. In order to prevent the invasion, it is critical to understand the invasive mechanisms of this species. However, few studies have been conducted on the allelopathic mechanisms of its invasion. In this study, we examined allelopathic effects of *P*. *virginica* on germination of seeds and growth of seedlings of four widely used lawn species. We found extensive allelopathic potential of *P*. *virginica* on other lawn species, which varied with species and developmental stage. While most effects of the extracts of *P*. *virginica* were inhibitory, some variables in some species were promoted by the addition of the extracts. The extracts of *P*. *virginica* significantly inhibited seed germination of *Agrostis matsumurae*. While the overall differences in seed germination rate of *Poa annua* were significant among treatments, difference between control and any of the treatments was not significant. The height of seedlings of *A*. *matsumurae* and *Cynodon dactylon* was significantly lower under the treatments of adding extracts of *P*. *virginica*. In contrast, growth of seedlings of *Festuca elata* and *P*. *annua* did not show significant differences among treatments. The root length of *A*. *matsumurae*, *C*. *dactylon* and *P*. *annua* was suppressed by the extracts of *P*. *virginica* whereas root length of *F*. *elata* was not affected. Aboveground biomass of *A*. *matsumurae* and *F*. *elata* was significantly higher than control, except for *F*. *elata* at the concentration of 50mg/mL, whereas aboveground biomass of *C*. *dactylon* and *P*. *annua* was reduced at higher concentrations of the extracts. Except for *A*. *matsumurae*, root biomass of the other three lawn species declined under the treatments with the extracts of *P*. *virginica*. Our results revealed that *P*. *virginica* had allelopathic potential on four lawn species and supported the theory of “novel weapons hypothesis”. Invasion by *P*. *virginica* in lawn can be moderated by selecting those species that are not affected or promotionally affected by it.

## Introduction

Biological invasion has become a great concern in the world. Many studies had expatiated the impacts of invasive species on native species, ecological environments, and incalculable economic loss from different aspects. Invasion threatens the biodiversity of the world [1, 2, 3, 4] because invasive species can reduce the richness and abundance of local species [5, 6, 7, 8] and change the genetic diversity of native species [9, 10]. Invasive species can change or disrupt ecosystem processes and functions [11, 12, 13, 14]. Biological invasion has brought a large amount of economic loss in the agriculture, forestry, livestock husbandry, and many other industries [15, 16, 17, 18, 19]. Plenty of studies have been conducted on the mechanisms of invasion and it has become a hotspot of ecology in the past few decades [20]. In 2002, Shea and Chesson generalized the mechanisms for the success of invasion into three aspects: physical environment, resource and natural enemy [21]. Ren and Zhang synthesized up to eight mechanisms for invasive success, including phenotypic plasticity in environmental tolerance and in resource allocation, competition for resource and utilization of resource, high growth or reproduction rate, allelopathy, evolutionary adaptation to physical environment, and evolutionarily increased competitive ability [22].

Successful invasion by a species may be a result of interaction between the invasive species and native species [23, 24]. Allelopathy is such a mechanism to help the exotic plants to invade new habitats successfully. Allelopathy means that plants can release some chemical substances into the surrounding environment, which can be inhibitory impacts on other plants directly or indirectly [25]. This is known as a mechanism or a theory of “novel weapons hypothesis” [26, 27, 28, 29], that is, allelochemical substances released by the non-native species in a new area are novel to native species, which aids their successful invasion [27]. For example, enhanced allelopathy and competitive ability of *Solidago canadensis* in its introduced range has been proven a key mechanism for its successful invasion [30]. Uddin *et al*. found *Phragmites australis*, one of the most aggressive invaders, had significant negative effects on germination and growth of a native species *Melaleuca ericifolia* [31]. *Acacia dealbata* produced an allelopathic substance, which influenced soil microbes, with bacteria being more sensitive to it than fungi [32]. *Ambrosia artemisiifolia* had allelopathic effects on some but not all native species [33]. Allelopathy of *Lonicera maackii* interacted with biotic and abiotic conditions [34]. Allelopathic substance might exist in different parts of the invasive species. For example, allelopathic substance in invasive *Alliaria petiolata* that impeded seed germination of *Brassica rapa* was in seeds [35], but aplotaxeney, an allelopathic substance in *Carduus nutans* and *Carduus acanthoides* that inhibited growth of lettuce, was found only in roots [36].

Invasion by alien species will remain a significant challenge in the future due to the globalization of human society [37]. Therefore, more studies are needed to understand the underlying mechanisms in order to prevent or control the invasion. *Plantago virginica* L. is a native annual species in North America and its introduction to China might be unintentionally [38]. *P*. *virginica* was first found in Jiangxi province of China in 1951 [39], but now it has spread to most of provinces in Southeast China. It is the dominant species in the area where it has invaded such as abandoned farmland, roadside and residential area, even forming nearly monoculture stands [40, 41]. It gains more attentions recently most likely because it has invaded into lawns in many cities. Invasion by *P*. *virginica* in lawns has obvious effects on the landscape and possible profound effects on the ecosystems. As a result of invasion by *P*. *virginica*, large amounts of money and labor are needed to remove them.

With the growing concerns on *P*. *virginica*, some studies have been conducted on this invasive species. Most of the researches were focusing on its biological or physiological characteristics, population dynamic and reproductive ecology [42, 43]. Among these results, some might help explain its invasive success. Fox example, population of *P*. *virginica* could be replaced after some time of establishment if no more disturbances happened [44]. *P*. *virginica* has an r-strategy of reproduction with more investment into reproductive organs [42], resulting in stronger reproductive ability than its native analog, *P*. asiatica *[45]*. *P*. *virginica* allocated more resource to reproductive growth than *P*. *asiatica under aluminium stress* [46] and chlorophyll fluorescence parameters exhibited stronger tolerance to *aluminium stress than P*. *asiatica* [47]. Warming could help enhance reproductive ability and thus invasive advantages of *P*. *virginica* [48].

Other possible mechanisms on how this species becomes successfully invaded is rarely studied and therefore poorly understood. It is crucial to thoroughly understand the mechanisms that facilitate its invasion to prevent or remove them, especially in lawn ecosystems where the invasion by *P*. *virginica* has caused economic loss. Here we conducted a study to investigate possible mechanism for successful invasion of *P*. *virginica* in lawn ecosystems. Our hypothesis is that *P*. *virginica* may have allelopathic potential on other species in lawns and this helps its establishment and becoming the dominant species in the lawns. Our study is also aimed to give some managerial suggestions to control *P*. *virginica* and maintain landscape.

## Materials and Methods

### 1. Preparation of the extracts of *P*. *virginica*

We took samples of whole individuals of *P*. *virginica* including roots in the lawn of Shanghai Institute of Technology (GPS coordinate: 31.165787 N, 121.421587 E) in May, 2013. No specific permission was required for those locations where we took the plant samples. And the field work did not involve endangered or protected species at this location. Plants were washed, dried, milled and sieved with 40–80 mesh. 200g milled powder was put into a 5000mL wide-mouth bottle, and 2000 mL of 50% ethanol solution were added into the bottle. After fully stirring, the sample was kept at room temperature for 48hrs to extract substance from *P*. *virginica* powder. The extraction solution was filtered by Buchner funnel. The filtrate was collected and concentrated with rotary evaporator (RE-52, Shanghai Zhenming Scientific Instruments Co., Ltd.). After ethanol was completely evaporated, the remaining extracted substance was re-dissolved in distilled water to a total volume of 2000mL. The extracts were stored in a 4°C refrigerator before application. The original concentration of the extracts was 100mg (milled powder)/mL. We further diluted the extracts to create five gradients of concentration: 0, 25, 50, 75 and 100mg/mL in our experiment treatments. Compounds contained in the extracts were analyzed with Agilent 7890–5975 gas chromatograph-mass spectrometer (GC-MS) system (Agilent Technologies, Inc.) after dissolved in trichloromethane. The compounds in the extracts were shown in S1 Table.

We didn’t use activated carbon in this study because it has been proven that activated carbon can also absorb substance other than allelochemicals, and change soil properties and soil microbial population structure [49, 50, 51, 52, 53].

### 2. Germination assay

Four commonly used lawn species, *Agrostis matsumurae* Hack. ex Honda, *Cynodon dactylon* (Linn.) Pers., *Festuca elata* Keng ex E. Alexeev and *Poa annua* L. were used in this study to investigate the allelopathic potential of *P*. *virginica* on these species.

100 seeds of each species were put onto the filter paper in a Petri dish that was 10cm in diameter. The extracts of *P*. *virginica* of five different concentrations, 0mg/mL, 25mg/mL, 50mg/mL, 75mg/mL and 100mg/mL, were added to the filter paper. The Petri dishes were kept in an incubator for seeds to germinate. The temperature was 24°C and the humidity was 80%, with light turned on for 12 hrs per day. Each concentration treatment of each species had 3 replicates, making a total of 60 samples (four species * five concentration * three replicates). We calculated the germination percentage when no new germination would occur.

### 3. Growth assay

Seeds of each species were sowed into sandy soil in a pot of 10cm in diameter. After germinating, 40 individuals were kept in the pot and the rest of individuals were removed. Five concentrations of the extracts of *P*. *virginica* that were same as in the germination experiment were applied into soils. Meanwhile the soils were watered with the same amount of tap water to keep the soils at a water content that was favorable for seedlings to grow. There were also 60 pots in total (four species * five concentration * three replicates). The pots were held in a greenhouse with a controlled temperature of 24°C and humidity of 80%. After growing for 2 weeks, the height of plants was measured. Then plants were removed from the soils and washed for measurements of root length. The plants were then dried at 60°C to a constant weight to determine the aboveground and root biomass.

### 4. Statistical analysis

One-way analysis of variance (ANOVA) and post-hoc Fisher tests were used to analyze the significance of the differences in germination rate, growth and biomass of seedlings among treatments. We tested the normality and homogeneity of variance before conducting ANOVA analysis. All of the statistical analysis was performed with Statistic 6.0.

## Results

### 1. The effects of the extracts of *P*. *virginica* on seed germination rate of four lawn species

**Fig 1:**
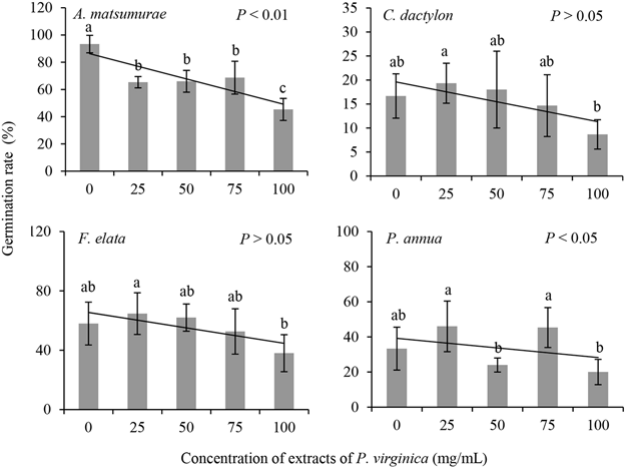
The effects of extracts of *P*. *virginica* on seed germination rate of four lawn species.

The extracts of *P*. *virginica* had significant effects on the germination rates of *A*. *matsumurae* and *P*. *annua*, but not on those of the rest two species (Table 1; Fig 1). The germination rate of *A*. *matsumurae* was 51.4% lower than control at the concentration of 100mg/mL.

**Table 1:** One-way analysis of variance (ANOVA) of allelopathic effects of *P*. *virginica* on seed germination, growth of seedlings, and biomass of seedlings of four lawn species.

Variables	Species	*df*	*F*	*P*
Germination rate	*A*. *matsumurae*	4, 10	13.13	< 0.01**
	*C*. *dactylon*	4, 10	1.70	0.23
	*F*. *elata*	4, 10	1.90	0.19
	*P*. *annua*	4, 10	3.84	< 0.05*
Seedling height	*A*. *matsumurae*	4, 10	22.71	< 0.01**
	*C*. *dactylon*	4, 10	6.46	< 0.01**
	*F*. *elata*	4, 10	0.66	0.63
	*P*. *annua*	4, 10	0.76	0.57
Root length	*A*. *matsumurae*	4, 10	8.74	< 0.01**
	*C*.*dactylon*	4, 10	4.24	< 0.05*
	*F*. *elata*	4, 10	1.35	0.32
	*P*. *annua*	4, 10	13.99	< 0.01**
Aboveground biomass of seedlings	*A*. *matsumurae*	4, 10	19.98	< 0.01**
	*C*. *dactylon*	4, 10	43.66	< 0.01**
	*F*. *elata*	4, 10	296.44	< 0.01**
	*P*. *annua*	4, 10	35.88	< 0.01**
Root biomass of seedlings	*A*. *matsumurae*	4, 10	0.89	0.51
	*C*. *dactylon*	4, 10	14.92	< 0.01**
	*F*. *elata*	4, 10	12.11	< 0.01**
	*P*. *annua*	4, 10	21.31	< 0.01**

The level of the significant difference was marked with “*” and “**”.

### 2. The effects of the extracts of *P*. *virginica* on the growth of seedlings of four lawn species

**Fig 2:**
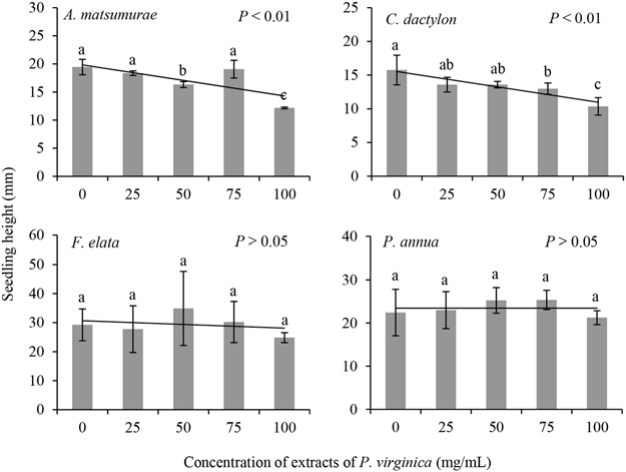
The effects of extracts of *P*. *virginica* on seedling height of four lawn species.

The growth of seedlings, i.e., the height of seedlings of *A*. *matsumurae* and *C*. *dactylon* was significantly suppressed by adding extracts of *P*. *virginica* (Table 1; Fig 2). For example, at the concentration of 100mg/mL, the height of seedlings of *A*. *matsumurae* and *C*. *dactylon* decreased by 37.1% and 34.2%, respectively compared with control. The inhibition by the extracts of *P*. *virginica* was aggravated with the increase of the concentration of the extracts with an exception at the concentration of 75mg/mL for *A*. *matsumurae*. However, the height of seedlings of *F*. *elata* and *P*. *annua* did not show any difference between control and any treatment with extracts of *P*. *virginica* (Table 1; Fig 2).

**Fig 3:**
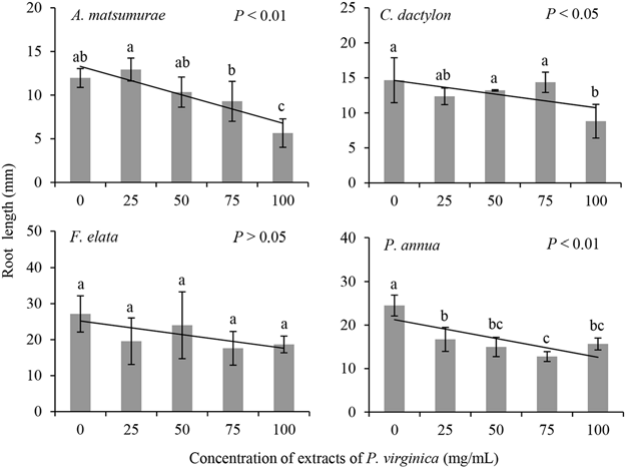
The effects of extracts of *P*. *virginica* on root length of four lawn species.

Unlike the growth of seedlings, growth of roots of *P*. *annua*, as indicated by root length, was significantly influenced by adding the extracts of *P*. *virginica* (Table 1; Fig 3). Root length of the other three species showed very similar patterns with those of seedling height. That is, root length of *A*. *matsumurae* and *C*. *dactylon* was significantly shorter under treatments of extract addition, but the treatments did not affect root growth of *F*. *elata*. Specifically, the effects of the extracts on root length of *A*. *matsumurae* and *C*. *dactylon* were only significant at the concentration of 100mg/mL.

### 3. Biomass of seedlings of four lawn species as affected by of the extracts of *P*. *virginica*

**Fig 4:**
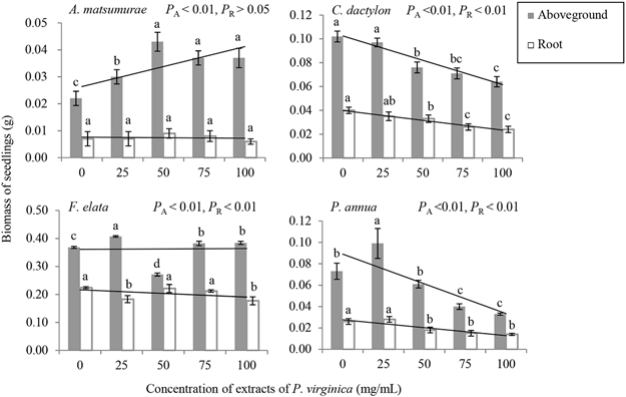
The effects of extracts of *P*. *virginica* on aboveground biomass and root biomass of seedlings of four lawn species. *P* values (significant level) of aboveground and root biomass were abbreviated as *P*_A_ and *P*_R_, respectively.

The extracts of *P*. *virginica* had significant effects on aboveground biomass of all four species, either promotional or inhibitory (Table 1; Fig 4). In detail, aboveground biomass of *A*. *matsumurae* and *F*. *elata* was higher than control, except for *F*. *elata* at the concentration of 50mg/mL. In contrast, aboveground biomass of *C*. *dactylon* and *P*. *annua* was significantly reduced from the concentration of 50 mg/mL and 75mg/mL, respectively compared with control.

The extracts of *P*. *virginica* significantly affected root biomass of *C*. *dactylon*, *F*. *elata* and *P*. *annua*, but had no significant effects on that of *A*. *matsumurae* (Table 1; Fig 4). In those three species whose root biomass was significantly reduced by addition of the extracts, root biomass declined with increase of the concentration of the extracts, but those for *F*. *elata* at the concentration of 50 mg/mL and 75 mg/mL were not significantly different from control.

## Discussion

### 1. Different responses of four lawn species to the extracts of *P*. *virginica*

In this study, we observed extensive allelopathic potential of invasive *P*. *virginica* on four lawn species. However, the effects of the extracts of *P*. *virginica* on these species were diverse, varying with species or different developmental stages. Seed germination, height of seedlings and root length of *A*. *matsumurae* were all suppressed by adding the extracts of *P*. *virginica*. However, root biomass of *A*. *matsumurae* did not change among treatments. Aboveground biomass of this species was even stimulated by the extracts. Growth of seedlings, in terms of seedling height and root length, and biomass of both aboveground and belowground of *C*. *dactylon* were all inhibited by application of the extracts of *P*. *virginica*, but germination rate of its seeds was not affected. Germination rate of seeds and growth of seedlings, including height of seedlings and length of roots of *F*. *elata* did not show any significant difference between control and treatments with the extracts of *P*. *virginica*. On the other hand, aboveground biomass of *F*. *elata* was enhanced and belowground biomass was reduced under treatments, with some exception at certain concentration of the extracts for both aboveground and belowground biomass. Root length and biomass of both aboveground and belowground of *P*. *annua* were significantly lower under treatments with the extracts of *P*. *virginica*, whereas height of seedlings did not change significantly among treatments. No significant difference was found in germination rate of *P*. *annua* between control and any of the other four concentrations of the extracts although the overall differences among the treatments were significant. In brief, most responses of four lawn species to the addition of the extracts of *P*. *virginica* were inhibitory, but there were some promotional responses or unchanged parameters, depending on species, developmental stages of plants, or different parts of the individuals, indicating the diverse interaction between invasive *P*. *virginica* and other species.

### 2. Possible causes for allelopathic potential of invasive *P*. *virginica* on other species

While we have found some allelopathic potential of invasive *P*. *virginica* on the other species, it is hard to specify which compound(s) is (are) responsible for the inhibitory or promotional effects because we used the extracts of whole plants of *P*. *virginica*. Actually potential allelopathic substance is diverse. It could be only one compound or several compounds together to inhibit the germination of seeds or growth of seedlings of the other species. Silva *et al*. found that it was a kind of secondary metabolites in *Carduus*, aplotaxene that had allelopathic effects [37]. In a study on allelopathy of *Centaurea maculosa* by Inderjit *et al*., (±)-catechin was responsible for allelopathic potential [54]. However, there were two substances, apigenin and chlorogenic acid, in *Lonicera maackii* that functioned as allelopathic substance [55].

It is also possible that the content of some extant substance has changed largely after a species invaded into a new area and it is the increased substance that acts as allelopathic chemical. Liu *et al*. found that ethyl phydroxybenzoate, p-hydroxybenzaldehyde, dhurrin, and apigenin of *Sorghum halepense* in invaded area in China had allelopathic effects, but their concentrations have changed a lot compared with those reported by Czarnota *et al*. in 2003 [56, 57] in their native region in the U.S. The changes in chemical composition or in content of some compounds that favor invasion of plants might be caused by evolutional changes that the invasive plants have undergone in the new region where they have invaded [58, 59]. We also found some changes in content of total flavonoids and polysaccharides of *P*. *virginica* between invaded area and its original habitat (unpublished data). For example, with the rutin as a standard substance, we analyzed the content of total flavonoids in the leaves of the native and invasive *P*. *virginica*. The content of total flavonoids of native and invasive *P*. *virginica* was 1.51% and 2.56%, respectively. Another possibility is that invasive species can release a totally new substance that does not exist when grown in its original environment. Further studies are needed to separate the specific substance that causes allelopathic potential of *P*. *virginica*.

### 3. Implications of allelopathic potential of invasive *P*. *virginica* on other species

As shown above, some variables of some species were not affected or even promoted by use of the extracts of *P*. *virginica*. In addition, so far most noticeable invasion by *P*. *virginica* occurs in lawn ecosystems. This provides an opportunity for the managers of the lawn engineering to select those lawn species that are insensitive to the extracts of *P*. *virginica* in order to avoid invaded by *P*. *virginica*. For example, from our study, at earlier stage of growth, i.e., seed germination, *C*. *dactylon* and *F*. *elata* would not been affected by *P*. *virginica*. During later stage of seedling growth, *F*. *elata* was less affected by *P*. *virginica*. Both seedling height and root length of *F*. *elata* remained unchanged under treatments of extract addition, but its biomass was significantly affected. Considering the biomass, *A*. *matsumurae* would be better choice for lawns because root biomass was not influenced and aboveground biomass was stimulated by the extracts of *P*. *virginica*. However, as we can see from the above recommendations, different conclusions may be drawn when different variables are taken into account. A comprehensive evaluation is necessary to select a better species for lawns from the perspective of preventing invasion by *P*. *virginica*.

Most allelopathic potential of invasive *P*. *virginica* on other species is inhibitory but we did find some promotional effects. Fox example, aboveground biomass of *A*. *matsumurae* and *F*. *elata* was higher under the treatments with addition of the extracts of *P*. *virginica*. This suggests that some native species derive benefits from the invasion of *P*. *virginica* [60]. We may also pay attention to the promotional role of an invasive species because we usually focus on their inhibitory effects and these promotional effects may be used to control them.

### 4. Other possible mechanisms for invasive success of *P*. *virginica*

*P*. *virginica* is known as a pioneer plant in its native habitats [40]. Pioneer plants usually have a great ability to change the physical and chemical properties of soils and soil microbial community composition and structure [61, 62, 63], which facilitates establishments of other species. However, in invaded area, this species may change physical and chemical properties and microbial community composition and structure of soils towards being unfavorable for other species to grow and reproduce [64, 65]. Our preliminary study on comparison of microbial composition and structure between invaded soils and non-invaded soils showed some differences in microbial community composition and structure (see S1 Text and S2 Table). For example, although the relative abundance of dominant taxa of both bacteria and fungi (i.e., Proteobacteria, Acidobacteria, Ascomycota, and Basidiomycota) had no significant differences, the relative abundance of some taxa of bacteria and fungi (e.g., Cyanobacteria, Lentisphaerae, Nitrospira, OP11, SR1, Spirochaetes, Blastocladiomycota, Chytridiomycota, Glomeromycota) changed significantly between invaded and non-invaded soils. These results indicate that the invasion of *P*. *virginica* has changed the microenvironment. The altered micro-environment, in turn, was more favorite for its successful invasion.

### 5. Possible values of invasive *P*. *virginica*

One species of *Plantago* in the U.S., *P*. *lanceolata*, was native in Europe. Recently its seeds were developed as health products. In China, many species of the same genus have historically and extensively been used as materials of Chinese medicine due to its medicinal functions [66, 67]. Lots of studies have reported active compounds in *P*. *asiatica* [68, 69, 70, 71, 72, 73], *P*. *major* and *P*. *lanceolata*. However, there is no report about active compounds in *P*. *virginica*. Therefore, we should amplify research in this area. After we know more about its active compounds, it is possible to utilize *P*. *virginica* for medical purpose or developing health products. At the same time we utilize it, we can also control its invasion.

## Supporting Information

S1 Table Components in the extracts of *P*. *virginica*. (DOCX)

S2 Table Comparison on microbial relative abundance (%) and multiple difference (log2) between invaded and non-invaded soils. (DOCX)

S1 Text Methods and preliminary results of microbial metagenomic sequencing in invaded and non-invaded soils. (DOCX)
