# Supplementary material for: Allelopathic Potential of Invasive Plantago virginica on Four Lawn Species
Source: PLoS One. 2015 Apr 27;10(4):e0125433. doi: 10.1371/journal.pone.0125433 (PMC4411108; doi:10.1371/journal.pone.0125433)
Supplement: S1 Table — (DOCX) [file pone.0125433.s001.docx]

**S1 Table.** **Components in the extracts of *P. virginica*.** We took 50mL original solution at a concentration of 100mg (milled powder)/mL and extracted it with 50mL trichloromethane in a conical flask. The extracts were collected with the separatory funnel. The extracts was then condensed to less than 5mL and filtered with Whatman paper. The filtrate was diluted with trichloromethane to 5mL. 1μL of the filtrate was injected into Agilent 7890-5975 gas chromatograph-mass spectrometer (GC-MS) system. The GC-MS was fitted with HP-INnowax capillary column (60m*0.25mm*0.25μm) and operated in the unsplit mode with helium as the carrier gas. The oven temperature started from 50℃ and was increased by 5℃ /min until 230℃. The temperature was held at 230℃ for 10 min. 25 kinds of component were detected. The relative content of 6, 10, 14-trimethyl-2-pentadecanone was the highest.

| **No.** | **Retention time** | **Component** | **Relative area (%)** |
| --- | --- | --- | --- |
| 1 | 10.877 | Dodecane | 0.24 |
| 2 | 11.987 | 1-dodecene | 0.57 |
| 3 | 15.978 | Tetradecane | 0.74 |
| 4 | 17.162 | 1-tetradecene | 1.85 |
| 5 | 17.572 | 3-methylene-tridecane | 0.47 |
| 6 | 18.526 | Pentadecane | 1.27 |
| 7 | 19.855 | 2-methyl-pentadecane | 0.45 |
| 8 | 20.144 | 3-methyl-pentadecane | 0.4 |
| 9 | 20.984 | Hexadecane | 1.99 |
| 10 | 22.126 | 1-hexadecene | 3.76 |
| 11 | 22.567 | 2,6,10,14-tetramethyl-pentadecane | 1.38 |
| 12 | 23.337 | Heptadecane | 2.04 |
| 13 | 24.064 | 1,2-benzenedicarboxylic acid diethyl ester | 0.69 |
| 14 | 25.585 | Octadecane | 0.82 |
| 15 | 26.675 | (E)-5-octadecene | 0.74 |
| 16 | 26.714 | 1-octadecene | 5.81 |
| 17 | 27.738 | Nonadecane | 0.42 |
| 18 | 29.626 | 2,7-dimethyl-naphthalene | 1.87 |
| 19 | 29.782 | Eicosane | 0.41 |
| 20 | 30.861 | (E)-5-eicosene | 0.26 |
| 21 | 32.373 | 6,10,14-trimethyl-2-pentadecanone | 7.14 |
| 22 | 33.894 | 1,6,7-trimethyl-naphthalene, | 0.52 |
| 23 | 34.054 | Hexadecanoic acid methyl ester | 0.82 |
| 24 | 34.191 | 3,3-dimethyl-biphenyl | 0.88 |
| 25 | 35.434 | Tricosane | 0.84 |
