# Supplementary material for: Allelopathic Potential of Invasive Plantago virginica on Four Lawn Species
Source: PLoS One. 2015 Apr 27;10(4):e0125433. doi: 10.1371/journal.pone.0125433 (PMC4411108; doi:10.1371/journal.pone.0125433)
Supplement: S2 Table — (DOCX) [file pone.0125433.s002.docx]

**S2 Table. Comparison on microbial relative abundance (%) and multiple difference (log2) between** **invaded and non-invaded soils.** Microbial relative abundance in invaded soil and non-invaded soil were abbreviated as “inv” and “non-inv”, respectively.

| **Microbe** | **Phylum** | **Invaded soils (%)** | **Non-invaded soils (%)** | **Multiple difference (log2 inv/non-inv)** |
| --- | --- | --- | --- | --- |
| Bacteria | Acidobacteria | 21.62178 | 23.75724 | -0.14 |
|  | Actinobacteria | 3.26820 | 2.44775 | 0.42 |
|  | BRC1 | 0.02934 | 0.02518 | 0.22 |
|  | Bacteria_incertae_sedis | 0.00587 | 0.00001 | 9.20* |
|  | Bacteroidetes | 8.68978 | 7.70587 | 0.17 |
|  | Chloroflexi | 3.15672 | 1.66205 | 0.93 |
|  | Cyanobacteria | 0.16429 | 0.07051 | 1.22* |
|  | Deferribacteres | 0.00001 | 0.00504 | -8.98* |
|  | Firmicutes | 0.44006 | 0.21657 | 1.02* |
|  | Gemmatimonadetes | 2.04776 | 3.30899 | -0.69 |
|  | Lentisphaerae | 0.00587 | 0.03526 | -2.59* |
|  | Nitrospira | 1.60183 | 3.49030 | -1.12* |
|  | OD1 | 0.66303 | 0.63964 | 0.05 |
|  | OP10 | 0.14082 | 0.10073 | 0.48 |
|  | OP11 | 0.02347 | 0.07051 | -1.59* |
|  | Planctomycetes | 4.00164 | 3.57089 | 0.16 |
|  | Proteobacteria | 42.30476 | 44.40695 | -0.67 |
|  | SR1 | 0.00001 | 0.02518 | -11.30* |
|  | Spirochaetes | 0.03521 | 0.07555 | -1.10* |
|  | TM7 | 0.21123 | 0.15110 | 0.48 |
|  | Verrucomicrobia | 7.97395 | 5.89776 | 0.44 |
|  | WS3 | 3.60852 | 2.33694 | 0.63 |
| Fungi | Ascomycota | 67.75561 | 64.54704 | 0.07 |
|  | Basidiomycota | 31.32027 | 29.74739 | 0.07 |
|  | Blastocladiomycota | 0.047089 | 0.00001 | 12.20* |
|  | Chytridiomycota | 0.470893 | 5.139373 | -3.45* |
|  | Glomeromycota | 0.141268 | 0.50813 | -1.85* |

“*” meant significant difference between invaded soils and non-invaded soils.
