# Supplementary material for: Allelopathic Potential of Invasive Plantago virginica on Four Lawn Species
Source: PLoS One. 2015 Apr 27;10(4):e0125433. doi: 10.1371/journal.pone.0125433 (PMC4411108; doi:10.1371/journal.pone.0125433)
Supplement: S1 Text — (DOCX) [file pone.0125433.s003.docx]

**S1 Text. Methods and preliminary results of microbial metagenomic sequencing in invaded and non-invaded soils.**

Soil sampling sites were the same as *P. virginica* was sampled. The turf was planted with *P. annua* since 2007. In the invaded sites, the dominant species was *P. virginica* and its coverage reached about 60% and coverage of *P. annua* was about 25%. In the non-invaded sites, *P. annua*, the dominant species, covered about 95%.

We selected three subplots in invaded and non-invaded region. Five soil cores of 5 cm diameter were collected randomly from each of three subplots to a depth of 20 cm. Upon arrival to the laboratory the soil samples were immediately frozen at -80℃.

The invaded soil pH and the non-invaded soil pH were determined with pH meter (Rex PHS-25, Shanghai INESA Scientific Instrument Co., Ltd., Shanghai, China). The mean were 6.97 and 6.91 respectively.

### Total DNA sequences were measured in a sequencing plate on a Roche 454 GS-FLX+ (454 Life Sciences, a Roche company, Branford, Connecticut, USA). 16S rRNA and ITS were used as tag sequences for bacteria and fungi, respectively. The sequence reads were separated based on their tag sequences for the soil samples. Sequences were clustered and assigned to operational taxonomic units (OTUs) using the QIIME implementation of cd-hit with a threshold of 97% pairwise identity. OTU numbers in each sample and sequence numbers in each OTU were analyzed by statistical analysis. Taxonomic information of each OTU was obtained by finding the nearest ancestor. Similar OTUs were clustered at the Phylum taxon. Relative abundance belonged to the same order of magnitude. The multiple difference was calculated as logarithmic quotient of relative abundance of the two types of soils. When the relative abundance value was 0, we used 0.00001 to calculate. Logarithmic values that were less than -1 or greater than 1 meant the significant difference in the multiple difference.

The preliminary results were shown in S2 Table. Although the relative abundance of dominant taxa of both bacteria and fungi (i.e., Proteobacteria, Acidobacteria, Ascomycota, and Basidiomycota) had no significant differences between invaded and non-invaded soils, some taxa of both bacteria and fungi (e.g., Cyanobacteria, Lentisphaerae, Nitrospira, OP11, SR1, Spirochaetes, Blastocladiomycota, Chytridiomycota, Glomeromycota) showed significant differences in the relative abundance between invaded and non-invaded soils. This indicated that the invasion of *P. virginica* could change the micro-environment, and the changed micro-environment, in turn, was more favorite for its successful invasion.
